# The FT_3_/FT_4_ Ratio as a Metabolic Marker of Frailty and Prognosis in Older Adults with Heart Failure

**DOI:** 10.3390/jcm14144840

**Published:** 2025-07-08

**Authors:** Chukwuma Okoye, Tessa Mazzarone, Filippo Niccolai, Alberto Finazzi, Emma Esposito, Giuseppe Bellelli, Agostino Virdis

**Affiliations:** 1 Department of Medicine and Surgery, University of Milano-Bicocca, 20126 Milano, Italy; a.finazzi15@campus.unimib.it (A.F.); e.esposito15@campus.unimib.it (E.E.);; 2 Fondazione IRCCS San Gerardo dei Tintori, 20900 Monza, Italy; 3 Geriatrics Unit, Department of Clinical and Experimental Medicine, University of Pisa, 56122 Pisa, Italy; tessa.mazzarone@phd.unipi.it (T.M.); filippo.niccolai9@gmail.com (F.N.); agostino.virdis@unipi.it (A.V.)

**Keywords:** frailty, biomarkers, heart failure, thyroid hormones, prognosis

## Abstract

**Background/Objectives:** Frailty is a key determinant of outcomes in older adults with heart failure (HF). The free triiodothyronine/free thyroxine (FT_3_/FT_4_) ratio has emerged as a promising frailty biomarker that reflects metabolic and systemic resilience. This study investigates its association with frailty, nutrition, muscle strength, inflammation, and one-year mortality in very old patients with HF. **Methods:** In this longitudinal, single-center study, we enrolled 193 older outpatients (mean age, 86.5 ± 6.1 years; 56% women) recently discharged after acute HF. All patients underwent physical examination, blood testing, and comprehensive geriatric assessment, including handgrip strength (HGS). Participants were stratified by FT_3_/FT_4_ ratio (<1.7 vs. ≥1.7). Associations with the Clinical Frailty Scale (CFS) were examined using multivariable linear regression. Spearman’s correlations assessed relationships with inflammatory and nutritional biomarkers. Cox regression evaluated the association with all-cause mortality. **Results:** Patients with a low FT_3_/FT_4_ ratio (31.1%) exhibited greater frailty (CFS: median [IQR], 6 [2] vs. 4 [3]; *p* = 0.020), poorer nutritional status (Mini Nutritional Assessment: 10 [4] vs. 12 [3]; *p* = 0.008), and lower HGS (mean ± SD, 16.8 ± 3.7 kg vs. 20.3 ± 4.8 kg; *p* = 0.002). An inverse association was identified between the FT_3_/FT_4_ ratio and frailty (adjusted β = −0.09; *p* = 0.019). Individuals with low FT_3_/FT_4_ also showed elevated inflammatory markers and had more than double the one-year mortality rate compared to those with higher ratios [HR 2.32 (95% CI, 1.24–4.34; *p* = 0.007)]. **Conclusions:** In very old adults recently hospitalized for HF, a lower FT_3_/FT_4_ ratio was associated with frailty, malnutrition, inflammation, and increased mortality, supporting its potential role as a marker of biological vulnerability.

## 1. Introduction

Heart failure (HF) represents a significant public health challenge worldwide due to its high prevalence, morbidity, and mortality rates [1,2]. The global burden of HF is increasing, with the prevalence projected to rise steeply as populations age, further straining healthcare systems.

Frailty, a dynamic and clinically identifiable condition that increases vulnerability to adverse events [3,4], is highly prevalent among older adults with HF. Prevalence estimates range from 19% to 52% in outpatient populations [5] and from 56% to 76% in hospitalized patients, increasing with age [6].

Both frailty and HF share underlying pathophysiological mechanisms, including inflammation, and muscle disorders, which together may exacerbate morbidity, mortality, and recurrent hospitalizations [6]. Although frailty is potentially reversible, progress in clinical management is hindered by the limited availability of diagnostic tools with adequate sensitivity, specificity, and predictive value [7]. Most current instruments for assessing frailty rely heavily on clinical judgment and the method of data collection, both of which are susceptible to operator variability and may compromise inter-rater reliability [8]. As a result, the search for reliable biomarkers to enhance diagnostic accuracy and enable more precise monitoring has become a key priority in the field [9]. However, despite significant efforts in recent years, our understanding of frailty biomarkers, their effectiveness, and their clinical utility remains inconclusive [10]. Thyroid hormones (TH), particularly the FT_3_/FT_4_ ratio, have recently emerged as potential biomarkers for frailty in various clinical settings [11,12,13]. This ratio reflects the efficiency of peripheral conversion of thyroxine (T_4_) to its biologically active form, triiodothyronine (T_3_), a process primarily mediated by type 1 deiodinase (DIO1) [14,15]. Deiodinases are enzymes expressed in several tissues, including the liver, skeletal muscle, and central nervous system—organs that are often compromised in frail individuals. In this context, reduced expression or activity of deiodinases may impair peripheral T_4_ -to- T_3_ conversion, leading to a relative decrease in thyroid hormone availability [11,13]. Such alterations may be driven by low-grade chronic inflammation, sarcopenia, and the cumulative burden of comorbidities commonly observed in frail HF patients [12]. Although alterations in thyroid function, particularly reduced FT_3_ levels, have been linked to adverse outcomes in older adults, existing evidence largely derives from studies conducted in acute care settings or cross-sectional analyses [11,14,16]. Limited attention has been given to the prognostic role of thyroid hormone dynamics in clinically stabilized older patients, particularly within transitional care contexts. Moreover, the integration of thyroid biomarkers with structured frailty assessments and longitudinal outcomes remains insufficiently explored, representing a critical gap in the literature.

The primary aim of this study is to assess the correlation between the FT_3_/FT_4_ ratio and markers of frailty, including nutritional status, muscle strength and inflammatory biomarkers. Additionally, we seek to address the prognostic value of the FT_3_/FT_4_ ratio in predicting one-year all-cause mortality among older outpatients with HF. By elucidating these associations, the study aims to enhance risk stratification and inform personalized management strategies for frail, older patients with HF.

## 2. Materials and Methods

This single-center, retrospective observational analysis based on a prospectively maintained registry enrolled patients aged 75 years and older who were evaluated in the Cardio-Geriatric Ambulatory Service between 1 January 2020 and 30 September 2022. All participants had been recently discharged from the Geriatric Unit of a tertiary care hospital (Azienda Ospedaliero-Universitaria Pisana, Pisa, Italy) with a diagnosis of acute decompensated HF (AHF). The diagnosis of acute HF was established during hospitalization according to the ESC Guidelines for the diagnosis and treatment of acute and chronic heart failure (2021). Diagnosis was based on the presence of typical clinical signs and symptoms (e.g., dyspnea, peripheral edema, pulmonary rales), elevated natriuretic peptides (NT-proBNP), and supportive imaging findings, including chest X-ray and bedside transthoracic echocardiography [17]. Patients who died during hospitalization or before the ambulatory assessment were excluded from the study. Additional exclusion criteria encompassed a known history of thyroid disease or the use of medications that could influence thyroid function, including amiodarone, methimazole, or thyroid hormone supplements.

### 2.1. Clinical and Laboratory Assessments

The Cardio-Geriatric Ambulatory Clinic operates twice weekly, staffed by a multidisciplinary team comprising an internal medicine consultant and a geriatrician (one per day), supported by two geriatric residents and a nurse. Patients recently discharged from the geriatric unit after an acute HF episode are systematically re-evaluated within 30 days [18].

All participants underwent a comprehensive geriatric assessment (CGA) [19], which included cognitive evaluation through the Short Portable Mental Status Questionnaire (SPMSQ) [20], assessments of dependence in basic and instrumental activities of daily living (BADL [21]/IADL [22]), and comorbidity burden using the Cumulative Illness Rating Scale Comorbidity Index (CIRS-c) [23]. Nutritional status was assessed using the Mini Nutritional Assessment-Short Form (MNA-SF) [24], and the body mass index (BMI) was calculated for each participant. Frailty was evaluated through the Clinical Frailty Scale (CFS) [25], which is routinely applied in our cardiogeriatric outpatient pathway. The choice of CFS was based on its strong validation in acute and post-acute heart failure settings, as demonstrated in large cohorts [8]. CFS also allows for rapid and reproducible evaluation within clinical workflows, minimizing missing data. The CFS was evaluated during the ambulatory assessment by two trained residents in geriatric medicine, using a standardized classification tree [26] to enhance consistency. Importantly, both assessors were blinded to thyroid hormone results, which were processed independently and not disclosed at the time of the frailty assessment. Additionally, we measured the patient’s muscle strength using the handgrip strength (HGS) test, which is a reliable measure of overall muscle function and an indicator of dynapenia [27]. The HGS test was performed with the dominant hand, and the highest score out of three consecutive measurements was recorded.

Cardiac function was assessed using focused cardiac ultrasound (FOCUS), following the principles of the focused assessed transthoracic echocardiography (FATE) protocol [28]. This included the evaluation of left ventricular ejection fraction (LVEF), myocardial dyskinesia, valve pathologies, and volume status indicators such as pericardial effusion and vena cava overload. Additionally, lung ultrasound and pleural effusion scoring were performed to assess pulmonary status [29].

### 2.2. Laboratory Measures

Routinary blood samples were collected on the first morning following the previous admission to the Geriatric Unit. Serum levels of thyroid-stimulating hormone (TSH), free triiodothyronine (FT_3_), and free thyroxine (FT_4_) were measured with immunohistochemistry (Ortho-Clinical Diagnostic, Amersham, UK). The normal ranges considered were 0.4–4 mIU/L for TSH, 2.7–5.7 ng/L for FT_3_, and 0.7–1.7 ng/dL for FT_4_. Additionally, levels of high-sensitivity C-reactive protein (Hs-CRP), N-terminal pro-B-type natriuretic peptide (NT-proBNP), albumin, uric acid, fibrinogen, serum creatinine, and ferritin were measured on the day before the ambulatory follow-up visit (see Supplementary Figure S1). In agreement with a previous study [13], patients were categorized as low FT_3_/FT_4_ or high FT_3_/FT_4_ using a cut-off of 1.7.

### 2.3. Statistical Analysis

Socio-demographic and clinical characteristics were analyzed using descriptive statistics. Categorical variables were reported with absolute frequencies and percentages, continuous variables with medians and interquartile ranges (IQR), or with means and standard deviations (SD). The normality of distribution was assessed for each variable using Shapiro’s test. Comparisons were performed with Student’s *t*-test, the Analysis of Variance (ANOVA) test, or the Kruskal–Wallis test for continuous variables, as appropriate, and with the χ^2^ test for categorical variables. Generalized linear models (GLMs) were performed to verify the association between FT_3_/FT_4_ (as a dependent variable) CFS, and HGS, adjusting for age, sex, and CIRS-c. A Spearman’s correlation matrix was applied to all continuous biochemical variables to assess the presence of significant correlations between FT_3_/FT_4_, inflammation, and nutritional biomarkers. After checking the proportional hazards assumption using Schöenfeld residuals, the hazard ratio (HR) and 95% CI of mortality were calculated to further evaluate the relationship between lower and higher levels of FT_3_/FT_4_ ratio and one-year-mortality as a univariable analysis and following adjustment for age, sex, and CIRS-c.

Statistical analyses were performed using RStudio software, Version 1.2.5001 (RStudio, Inc., Boston, MA, USA).

## 3. Results

### 3.1. Baseline Characteristics

The study flowchart is shown in Supplemental Figure S1. A total of 193 patients were included in the study, with a mean age of 86.5 years (SD = 6.1); 56.1% were female. The median interval between hospital admission and ambulatory reassessment was 17 days (IQR: 12–23). In 72% of cases, hospitalization was due to a primary diagnosis of acute HF (AHF), while in the remaining 28%, AHF developed as a secondary condition triggered by precipitating factors. These included infections such as pneumonia or urinary tract infections (12%), COPD exacerbations (7%), and anemia or dehydration (9%). No significant differences in the FT_3_/FT_4_ ratio were observed between patients with primary versus secondary AHF.

Among the participants, 60 patients (31.1%) had a low FT3/FT4 ratio (<1.7), while the remaining 133 had a high FT_3_/FT_4_ ratio (≥1.7). As shown in Table 1, no significant differences were observed between the two groups in sex distribution or the prevalence of chronic conditions, including hypertension, type 2 diabetes mellitus, chronic kidney disease, and chronic obstructive pulmonary disease.

**Figure 1:**
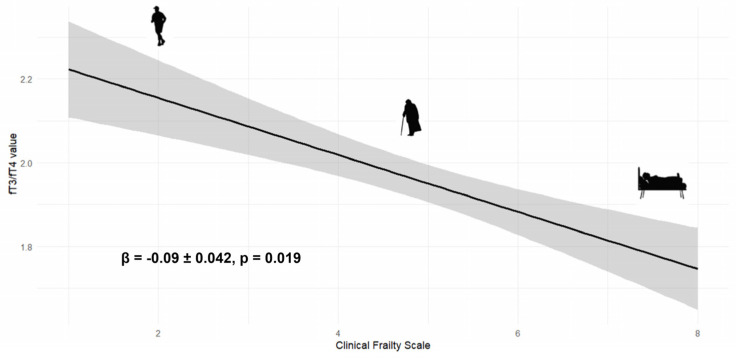
Inverse association between FT_3_/FT_4_ ratio and frailty severity. Multivariable linear regression model illustrating the relationship between the FT3/FT4 ratio (unitless) and the Clinical Frailty Scale (CFS; range 1–8). The regression line is plotted with a 95% confidence interval (gray shaded area). Higher CFS scores reflect greater frailty. Each symbol represents a stylized representation of increasing frailty stages.

The FT_3_/FT_4_ ratio showed a significant decreasing trend with increasing CFS values (*p* < 0.001, ANOVA), a relationship that remained significant in the multivariable linear regression model (adjusted β = −0.09 ± 0.042; *p* = 0.019; see Figure 1).

Patients with a low FT_3_/FT_4_ ratio were more likely to be frail compared to their counterparts (median CFS [IQR], 6 [2] vs. 4 [3]; *p* = 0.020). As shown in Table 1, patients in the low FT_3_/FT_4_ ratio group exhibited significantly worse performance across all CGA domains than others. Specifically, they were less independent in BADLs (median [IQR], 4 [5] vs. 5 [4]; *p* = 0.022) and IADLs (median [IQR], 1 [5] vs. 2 [5]; *p* = 0.047) and had worse cognitive function, as measured by the SPMSQ (median [IQR], 4 [3] vs. 3 [3]; *p* = 0.031).

### 3.2. Nutritional and Inflammatory Markers

Patients in the low FT3/FT4 ratio group demonstrated poorer nutritional status, with lower median MNA scores (median [IQR], 10 [4] vs. 12 [3]; *p* = 0.008), serum albumin levels (mean ± SD, 3.19 ± 0.51 g/dL vs. 3.42 ± 0.46 g/L; *p* < 0.001), and transferrin levels (mean ± SD, 175.1 ± 65.5 mg/dL vs. 204.2 ± 57.2 mg/dL; *p* < 0.001, see Table 1) compared with their counterparts. Handgrip strength was also lower in this group (16.8 ± 3.7 kg vs. 20.3 ± 4.8 kg; *p* = 0.002). The GLM revealed a significant positive association between FT_3_/FT_4_ ratio and handgrip strength (standardized β = 0.373 ± 0.017, *p* = 0.043).

Inflammatory markers were significantly elevated in patients with a low FT_3_/FT_4_ ratio. Hs-CRP levels were nearly twice as high in patients with lower ratios (mean 10.6 ± 9.7 mg/L vs. 5.8 ± 7.4 mg/L; *p* < 0.001), with similar trends observed for fibrinogen, ferritin, and ESR (see Table 1).

**Figure 2:**
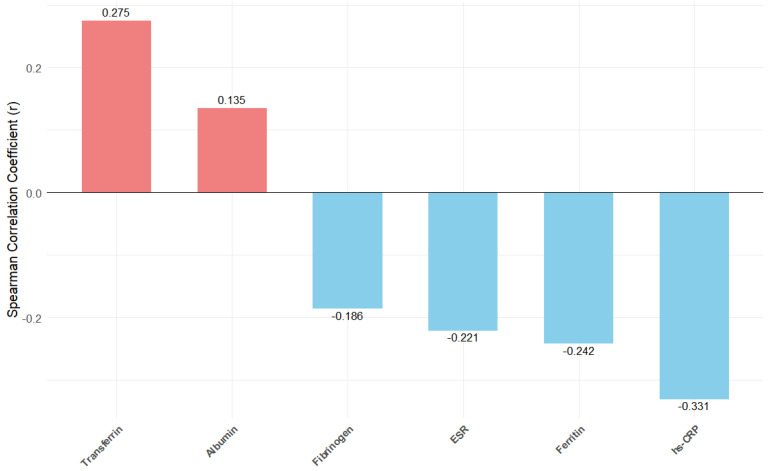
Spearman correlations between FT_3_/FT_4_ ratio and selected biomarkers. Bar plot showing correlation coefficients (r) between the FT_3_/FT_4_ ratio and markers of inflammation, nutrition, and function. Positive and negative correlations are shown in red and blue, respectively. Abbreviations: ESR = Eritrocyte Sedimentation Rate, Hs-CRP = High Sensitivity-C-Reactive Protein.

Spearman’s Rho correlation demonstrated a significant inverse relationship between the FT_3_/FT_4_ ratio and levels of ferritin, fibrinogen, and hs-CRP, whereas higher FT_3_/FT_4_ ratios were positively associated with transferrin and albumin levels (see Figure 2 and Supplementary Figure S3).

### 3.3. Cardiovascular Assessments

Cardiac assessments revealed no significant differences in median NT-proBNP levels between the groups. While not statistically significant, a higher prevalence of HF with preserved ejection fraction (HFpEF) was observed in the low FT_3_/FT_4_ group (56.1% vs. 40.2%), whereas HF with reduced ejection fraction (HFrEF) was less prevalent (29.8% vs. 41.2%; see Table 2).

### 3.4. Mortality Risk

**Figure 3:**
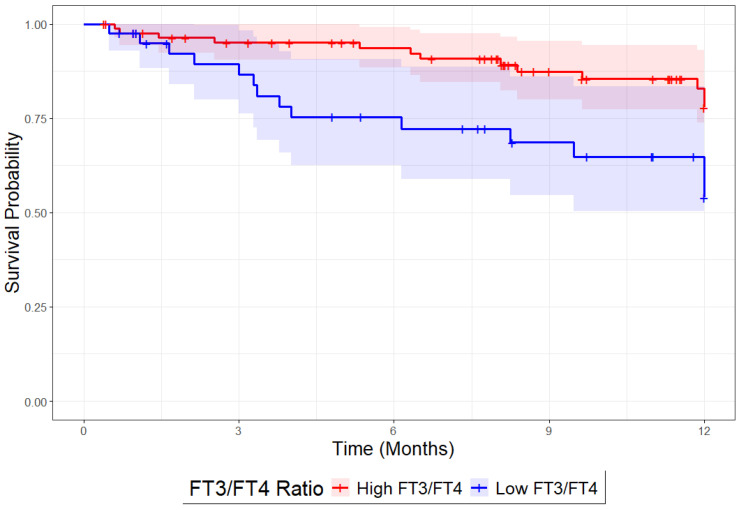
Kaplan–Meier survival curves for one-year mortality stratified by FT_3_/FT_4_ ratio. Patients are categorized into two groups based on the FT_3_/FT_4_ ratio: high (red line) and low (blue line).

Patients with a low FT_3_/FT_4_ ratio exhibited a significantly higher 1-year mortality risk compared to those with a high ratio (29.5% vs. 15.8%; *p* = 0.018). Kaplan–Meier survival curves underscored this disparity, revealing markedly higher mortality during the 1-year follow-up period in the low FT_3_/FT_4_ group (see Figure 3).

Multivariable Cox regression analysis confirmed these findings, with the low FT_3_/FT_4_ ratio group demonstrating more than double the mortality risk (HR: 2.35; 95% CI: 1.29–4.26; *p* = 0.004). This association remained robust after adjusting for age, sex, and comorbidities (adjusted HR: 2.32; 95% CI: 1.24–4.34; *p* = 0.007).

## 4. Discussion

In our cohort of very old outpatients recently discharged from the hospital after acute HF, an FT_3_/FT_4_ ratio below 1.7 was associated with more than a twofold increase in mortality risk compared to higher ratios, even after adjusting for multiple confounders. While the prevalence of chronic diseases was comparable between patients with high and low FT_3_/FT_4_ ratios, those with lower ratios exhibited greater frailty levels, higher rates of dynapenia, malnutrition, inflammation, and more pronounced cognitive and functional impairments. These findings suggest that the FT_3_/FT_4_ ratio can stratify a cohort of the oldest-old patients into two distinct clinical subgroups: those with a higher FT_3_/FT_4_ ratio, who exhibit greater fitness and resilience, and those with lower ratios, who are more vulnerable to adverse outcomes. Moreover, our analysis highlights that the FT_3_/FT_4_ ratio is independently associated with nutritional and inflammatory biomarkers, as well as with measures of physical strength, further underscoring its potential as a comprehensive marker of health status in this population.

Recently, the FT_3_/FT_4_ has been linked to adverse outcomes across multiple fields, including nephrology [30], cardiology [16], gastroenterology [31], diabetology [32], and oncology [33]. However, none of these studies have thoroughly evaluated the FT_3_/FT_4_ metabolic mechanisms or their relationship with frailty and its surrogates. Our study, therefore, broadens the current scope of the literature by confirming previous findings [13] and demonstrating FT_3_/FT_4_ as a surrogate marker of frailty, namely a condition of reduced physiological reserves, decreased resistance to stressors, and enhanced vulnerability to poor health outcomes, such as diseases, disability, falls, institutionalization, and death.

Although not statistically significant, we observed a difference in HF subtypes between FT_3_/FT_4_ groups, with patients having low FT_3_/FT_4_ levels showing a higher prevalence of HFpEF. This aligns with prior research linking thyroid dysfunction, particularly low FT_3_, to altered myocardial remodeling and diastolic dysfunction, which are hallmarks of HFpEF [34]. Studies have shown that reduced FT_3_ levels correlate with impaired left ventricular relaxation and increased myocardial stiffness, both of which contribute to the development of HFpEF [35]. This relationship is further supported by data indicating that thyroid hormones directly influence cardiac structure and function [36]. Low FT_3_ levels, for instance, have been associated with reduced cardiac output and increased peripheral vascular resistance, compounding the effects of age-related changes in myocardial composition. The presence of low-grade inflammation and oxidative stress in older adults exacerbates these abnormalities, contributing to the development of HFpEF as a geriatric syndrome [37]. As a fact, the interplay between non-cardiac comorbidities, such as chronic kidney disease and diabetes, further amplifies the risk of HFpEF in individuals with thyroid dysfunction. These chronic disorders foster a pro-inflammatory state that promotes myocardial fibrosis and stiffening, creating a substrate for HFpEF in patients with low thyroid hormone levels. Collectively, these findings highlight the intricate pathophysiological links between thyroid hormone deficiencies, chronic diseases, and HFpEF [37].

Furthermore, our findings suggested a dysregulated inflammatory response in patients with a low FT_3_/FT_4_ ratio, as inflammatory markers showed the strongest correlations among those tested. This underscores the potential role of inflammaging—a syndrome marked by chronic, low-grade inflammation arising from prolonged antigenic exposure and excess adipose tissue [38]—in impairing FT_3_/FT_4_ conversion. Inflammaging reflects cumulative changes in the innate immune system, driven by persistent immune activation and metabolic stress, ultimately contributing to thyroid hormone dysregulation [39].

Notably, the FT_3_/FT_4_ ratio demonstrated an independent association with nutritional status, as well as with transferrin and albumin levels, which are critical biomarkers of malnutrition and impaired hepatic function. Furthermore, a direct correlation between the FT_3_/FT_4_ ratio and handgrip strength—a well-established indicator of overall health, cardiovascular event risk, and mortality—was observed, in line with previous studies [40,41]. These findings suggest that a low FT_3_/FT_4_ ratio may be implicated in diminished muscle function and mass, consistent with sarcopenic and malnourished states. Indeed, muscle gene expression is controlled and regulated in a T3-dependent fashion [42]. Patients with hypothyroidism frequently experience proximal muscle wasting, fatigue, exercise intolerance, and muscle cramps. In hypothyroid myopathy, type II muscle fibers are more commonly affected by atrophy. This pattern may be explained by impaired mitochondrial oxidative metabolism resulting from thyroid hormone deficiency, which particularly impacts type II fibers due to their greater dependence on mitochondrial function compared to type I fibers. DIO2 locally converts T4 to active T3 in skeletal muscle. Therefore, the observed relationship between reduced FT_3_/FT_4_ levels and lower handgrip strength provides further evidence of impaired peripheral deiodination, which plays a central role in muscle and metabolic homeostasis [43].

Taken together, our findings support the hypothesis that peripheral thyroxine deiodination, as reflected by the FT_3_/FT_4_ ratio, may serve as a biomarker of healthy versus unhealthy aging. Individuals with higher FT_3_/FT_4_ ratios—irrespective of chronological age—exhibited greater physiological reserve, superior functional performance, and a lower risk of adverse outcomes, suggesting higher intrinsic capacity.

Moreover, this study extends current knowledge by examining the FT_3_/FT_4_ ratio within a real-world cohort of very old adults who were clinically stabilized following hospitalization for AHF. Unlike previous cross-sectional investigations, our design incorporated a comprehensive, multidomain assessment of frailty and a longitudinal follow-up at one year, providing dynamic insights into the prognostic significance of thyroid hormone imbalance. Collectively, these data underscore the potential utility of the FT_3_/FT_4_ ratio as an early indicator of biological vulnerability in transitional care settings.

A major strength of our study lies in its real-world, post-acute setting, where a comprehensive geriatric assessment, including cognitive, functional, nutritional, and cardiovascular evaluations, was systematically applied to a well-characterized cohort of the oldest-old. This enables a nuanced interpretation of FT_3_/FT_4_ levels in the broader context of frailty and physiological reserve.

Nevertheless, some limitations should be acknowledged. The single-center design and relatively small sample size may limit the generalizability of our findings, warranting validation in larger, multicenter studies. Additionally, a temporal discrepancy exists in the collection of biomarkers and functional assessments: thyroid hormones were measured upon hospital admission, while inflammatory and nutritional parameters were obtained approximately one to three weeks later during follow-up. While this may weaken associations with acute-phase markers, we also included more stable indicators of chronic inflammation—such as ESR, fibrinogen, and ferritin—which are less susceptible to short-term fluctuations and better reflect underlying biological vulnerability. In addition, several elements support the robustness of this timing. First, alterations in thyroid hormones during acute illness (e.g., low FT_3_ or reduced FT_3_/FT_4_ ratio) are known to persist beyond the acute phase, particularly in older or frail patients, with full recovery sometimes requiring up to 6–8 weeks [44,45]. Second, rather than representing a limitation, the early timing of measurement may enhance the clinical utility of FT_3_/FT_4_ ratio as a risk stratification tool, as it provides prognostic insight during the transition to post-acute care. Since the ratio reflects chronic pathophysiological mechanisms—such as inflammation, undernutrition, and sarcopenia—its reduction may identify vulnerable patients regardless of transient illness severity.

Finally, the absence of standardized measures of acute illness severity during hospitalization (such as APACHE II or SOFA scores) represents a limitation, as it precludes a more granular adjustment for acute physiological stress. However, this reflects the nature of the clinical setting, where high-intensity monitoring is not routinely performed. While this may introduce residual confounding, the exclusive inclusion of clinically stabilized patients and the use of post-acute frailty assessments were designed to mitigate its impact.

## 5. Conclusions

Our findings confirm that a low FT_3_/FT_4_ ratio is a reliable biomarker of frailty, consistently associated with dynapenia, malnutrition, inflammation, and both cognitive and functional decline. Given its low cost, clinical accessibility, and biological plausibility, the FT_3_/FT_4_ ratio may represent a valuable tool to assist clinicians in identifying older HF patients at heightened risk for poor outcomes. Its straightforward interpretation makes it especially useful even for those less familiar with comprehensive frailty assessments, ultimately supporting more personalized, risk-adapted management strategies.

## Figures and Tables

**Table 1:** Characteristics of the study population according to FT_3_/FT_4_ levels.

	All Patients N = 193	FT_3_/FT_4_ ≥ 1.7 N = 133	FT_3_/FT_4_ < 1.7 N = 60	*p*-Value
Sex (F)	119 (56.1)	73 (54.8)	35 (58.8)	0.636
Age (mean, SD)	86.5 (6.1)	86.2 (6.8)	87.1 (4.9)	0.585
BADL (median, IQR)	5 (5)	5 (4)	4 (5)	0.022
IADL (median, IQR)	2 (5)	2 (5)	1 (5)	0.047
CFS (median, IQR)	5 (3)	4 (3)	6 (2)	0.020
SPMSQ (median, IQR)	3 (4)	3 (3)	4 (3)	0.031
MNA (median, IQR)	11 (3)	12 (3)	10 (4)	0.008
Handgrip test, Kg, (mean, SD)	19.7 (4.5)	20.3 (4.8)	16.8 (3.7)	0.002
Females (mean, SD)	12.3 (7)	15.8 (4.9)	14.1 (2.3)	0.06
Males (mean, SD)	20.1 (10.3)	25.8 (7.8)	17.3 (3.9)	<0.001
CIRS-c (mean, SD)	3 (2)	3 (2)	3 (2)	0.81
Hypertension (%)	134 (66.3)	83 (63.1)	43 (72.2)	0.188
T2DM (%)	56 (27.7)	34 (26.1)	18 (30.5)	0.503
COPD (%)	54 (26.7)	66 (27.6)	15 (25.0)	0.679
CAD (%)	33 (16.4)	21 (16.3)	10 (16.7)	0.943
Stroke (%)	22 (11.0)	15 (11.5)	4 (10.0)	0.74
CKD (%)	73 (36.5)	53 (41.1)	17 (28.2)	0.069
AF (%)	114 (56.7)	81 (62.8)	21 (35.8)	0.020
TSH, mIU/L	1.97 (2.20)	2.02 (2.25)	1.87 (2.01)	0.699
Hs-CRP, mg/L (mean, SD)	7.8 (8.78)	5.8 (7.4)	10.6 (9.7)	<0.001
Fibrinogen, mg/dL (mean, SD)	461 (172)	439 (158)	495 (187)	0.003
Ferritin, ng/mL (mean, SD)	441.7 (798)	323.9 (465)	604 (1085)	<0.001
Transferrin, mg/dL (mean, SD)	192 (62.4)	204.2 (57.2)	175.1 (65.5)	<0.001
Albumin, g/dL, (mean, SD)	3.32 (0.49)	3.42 (0.46)	3.19 (0.51)	<0.001
ESR, mm/h, (mean, SD)	61.8 (36.2)	55.9 (35.6)	69.7 (35.8)	<0.001
Creatinine, mg/dL, (mean, SD)	1.44 (0.68)	1.40 (0.55)	1.46 (0.73)	0.28
NT-proBNP, pg/mL, (mean, SD)	1991 (3380)	1860 (3669)	2221 (1951)	0.966
Uric Acid (mg/dL), (mean, SD)	6.99 (2.85)	6.8 (2.6)	7.2 (3.1)	0.205

Data are expressed as mean (standard deviation) and number (%), as appropriate. Abbreviations: BADL = Basic Activities of Daily Living, IADL = Instrumental ADL, CFS = Clinical Frailty Scale, SPMSQ = Short Portable Mental Status Questionnaire, TSH = thyroid-stimulating hormone; CIRS = Cumulative Illness Rating Scale, T2DM = Type 2 Diabetes Mellitus, COPD = Chronic Obstructive Pulmonary Disease, CAD = Coronary Artery Disease, CKD = Chronic Kidney Disease, HS-CRP = High Sensitivity-C-Reactive Protein, ESR = Erythrocyte Sedimentation Rate.

**Table 2:** Heart failure-related features and biomarkers of the population according to low and high FT_3_/FT_4_ ratio.

	All Patients N = 193	FT_3_/FT_4_ > 1.7 N = 133	FT_3_/FT_4_ < 1.7 N = 60	*p*-Value
NT-proBNP, pg/mL (median, IQR)	1991 (3380)	1860 (3669)	2221 (1950)	0.966
Serum Iron, µg/dL (median, IQR)	43 (26.5)	44 (23)	43 (31)	0.154
EF (%)	52.5 (18)	55 (14.7)	51.5 (11.0)	0.106
HFrEF, n (%)	57 (37.0)	40 (41.2)	17 (29.8)	0.159
HFmrEF, n (%)	26 (16.8)	18 (18.6)	8 (14.0)	
HFpEF, n (%)	71 (46.1)	39 (40.2)	32 (56.1)	
LIS, mm (mean, SD)	12 (3.3)	11.9 (3.2)	12.4 (3.8)	0.493
PAPS, mm (median, IQR)	42.5 (13.1)	40 (11)	45 (14.2)	0.787
TAPSE, mm (median, IQR)	19.7 (3.5)	19.6 (3.7)	19.8 (3.1)	0.684
HR per minute (median, IQR)	70 (10)	70 (10)	75 (12)	0.814
SBP, mmHg (median, IQR)	124.1 (20)	123 (22.0)	126 (15.6)	0.513
DBP, mmHg (median, IQR)	68.9 (10.9)	67.8 (11.3)	71.3 (9.9)	0.077

Abbreviations: NT-proBNP = N-terminal pro–B-type natriuretic peptide; TnHS = high-sensitivity troponin; Serum Iron = serum iron concentration; EF = ejection fraction; HFrEF = heart failure with reduced ejection fraction (EF < 40%); HFmrEF = heart failure with mildly reduced ejection fraction (EF 40–49%); HFpEF = heart failure with preserved ejection fraction (EF ≥ 50%); LIS = inferior vena cava longitudinal index in systole; PAPS = pulmonary artery systolic pressure; TAPSE = tricuspid annular plane systolic excursion; HR = heart rate; SBP = systolic blood pressure; DBP = diastolic blood pressure.

## Data Availability

The data supporting the findings of this study are available from the corresponding author upon reasonable request.

## Supplementary Materials

The following supporting information can be downloaded at https://www.mdpi.com/article/10.3390/jcm14144840/s1: Figure S1: Timeline of in-hospital and ambulatory assessments. Figure S2: Study flowchart. Figure S3: Spearman correlation matrix between FT_3_/FT_4_ and functional, inflammatory, nutritional markers.

## Abbreviations

The following abbreviations are used in this manuscript:

ADL	Activities of Daily Living
AF	Atrial Fibrillation
BADL	Basic Activities of Daily Living
BMI	Body Mass Index
CFS	Clinical Frailty Scale
CGA	Comprehensive Geriatric Assessment
CKD	Chronic Kidney Disease
CIRS-c	Cumulative Illness Rating Scale-Comorbidity Index
COPD	Chronic Obstructive Pulmonary Disease
EF	Ejection Fraction
FT3	Free Triiodothyronine
FT4	Free Thyroxine
HF	Heart Failure
HFpEF	Heart Failure with Preserved Ejection Fraction
HFrEF	Heart Failure with Reduced Ejection Fraction
HGS	Handgrip Strength
Hs-CRP	High-Sensitivity-C-Reactive Protein
IADL	Instrumental Activities of Daily Living
LVEF	Left Ventricular Ejection Fraction
MNA-SF	Mini Nutritional Assessment-Short Form
NT-proBNP	N-terminal pro-B-type Natriuretic Peptide
SPMSQ	Short Portable Mental Status Questionnaire
TAPSE	Tricuspid Annular Plane Systolic Excursion
TnHS	High-Sensitivity Troponin
TSH	Thyroid-Stimulating Hormone
