# Supplementary material for: The FT3/FT4 Ratio as a Metabolic Marker of Frailty and Prognosis in Older Adults with Heart Failure
Source: J Clin Med. 2025 Jul 8;14(14):4840. doi: 10.3390/jcm14144840 (PMC12295841; doi:10.3390/jcm14144840)

**Figure S1:** Timeline of in-hospital and ambulatory assessments.
Schematic overview illustrating the temporal sequence of measurements. Thyroid hormone levels were obtained during the index hospitalization, while inflammatory, nutritional, and functional parameters were assessed 1 to 3 weeks later during post-acute ambulatory follow-up.

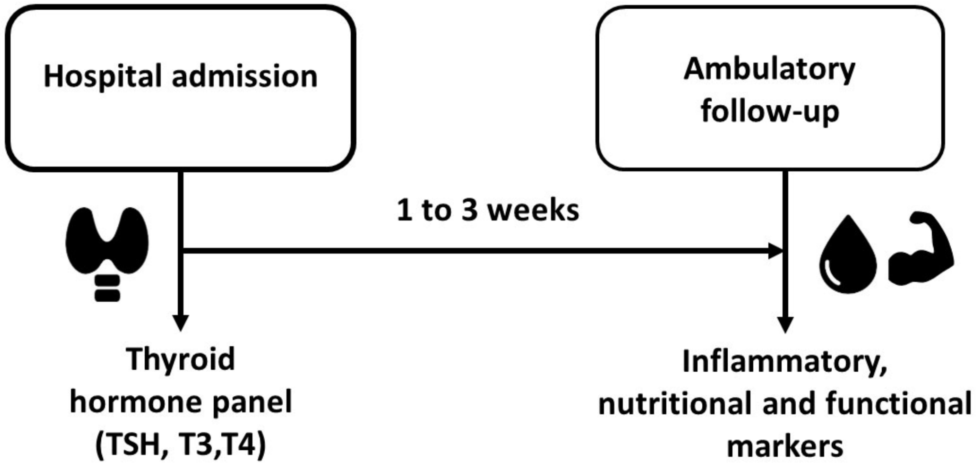


**Figure S2:** Study flowchart.

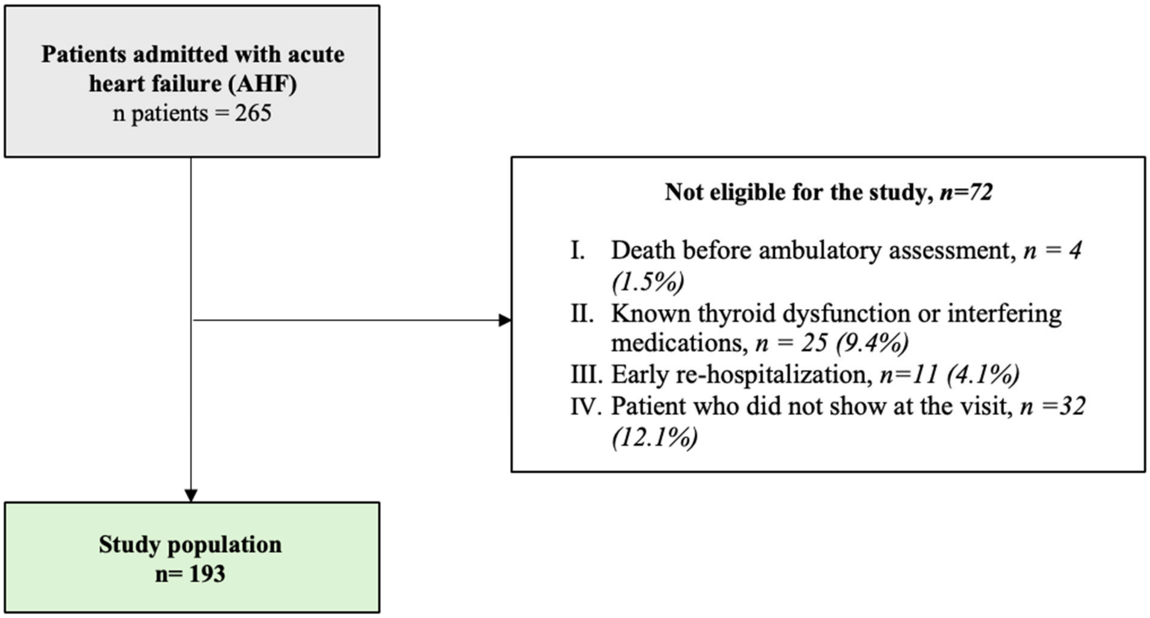

**Figure S3:** Spearman correlation matrix between $FT_3/FT_4$ and functional, inflammatory, nutritional markers.

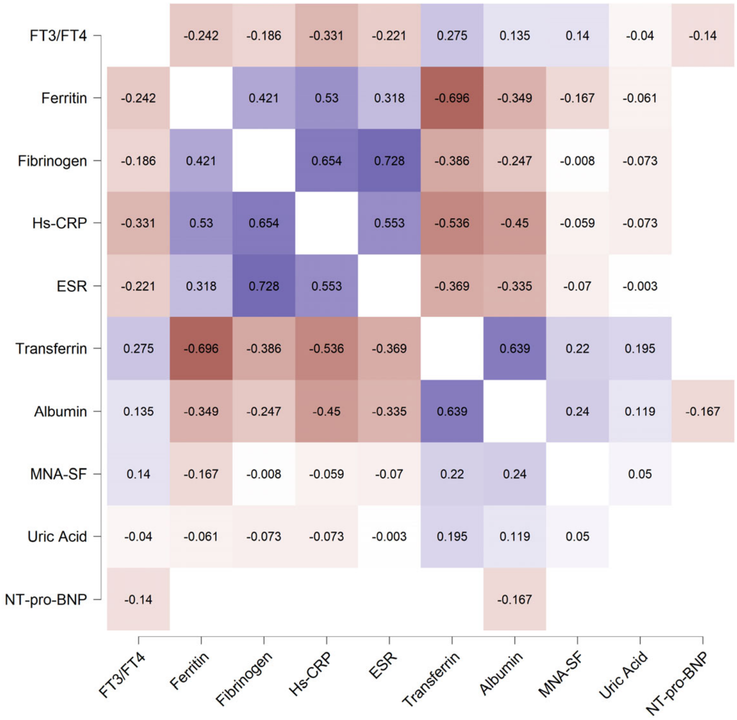

Supplement: Supplementary file 1 [file jcm-14-04840-s001.zip › jcm-3674590-supplementary.pdf]
